# CES2, ABCG2, TS and Topo-I Primary and Synchronous Metastasis Expression and Clinical Outcome in Metastatic Colorectal Cancer Patients Treated with First-Line FOLFIRI Regimen

**DOI:** 10.3390/ijms150915767

**Published:** 2014-09-05

**Authors:** Nicola Silvestris, Giovanni Simone, Giulia Partipilo, Emanuela Scarpi, Vito Lorusso, Anna Elisabetta Brunetti, Evaristo Maiello, Angelo Paradiso, Anita Mangia

**Affiliations:** 1 Medical Oncology Unit, National Cancer Research Centre—Istituto Tumori “Giovanni Paolo II”, Viale Orazio Flacco 65, 70124 Bari, Italy; E-Mails: vitolorusso@inwind.it (V.L.); brunetti.ae@gmail.com (A.E.B.); 2 Pathology Department, National Cancer Research Centre—Istituto Tumori “Giovanni Paolo II”, Viale Orazio Flacco 65, 70124 Bari, Italy; E-Mail: g.simone@oncologico.bari.it; 3 Functional Biomorphology Laboratory, National Cancer Research Centre—Istituto Tumori “Giovanni Paolo II”, Viale Orazio Flacco 65, 70124 Bari, Italy; E-Mails: giuliapartipilo@libero.it (G.P.); a.mangia@oncologico.bari.it (A.M.); 4 IRCCS Istituto Scientifico Romagnolo per lo Studio e la Cura dei Tumori (I.R.S.T.), 47014 Meldola, Italy; E-Mail: emanuela.scarpi@irst.emr.it; 5 Oncology Unit, IRCCS Casa Sollievo della Sofferenza, San Giovanni Rotondo (FG), Viale Cappuccini 1, 71013 San Giovanni Rotondo, Italy; E-Mail: e.maiello@operapadrepio.it; 6 Experimental Medical Oncology, National Cancer Research Centre—Istituto Tumori “Giovanni Paolo II”, Viale Orazio Flacco 65, 70124 Bari, Italy; E-Mail: a.paradiso@oncologico.bari.it

**Keywords:** irinotecan, leucovorin, 5-fluorouracil, carboxylesterase 2 (CES2), breast cancer resistance protein 2 (ABCG2), immunohistochemistry

## Abstract

Enzymatic activation of irinotecan (CPT-11) is due to carboxylesterase (CES), and its pharmacological behavior is influenced by drug resistance-related proteins. We previously reported that the clinical response and prognosis of metastatic colorectal cancer (mCRC) patients did not differ in tumors with different thymidylate synthase (TS) or topoisomerase-I (Topo-I) expression. Using immunohistochemistry (IHC), we evaluated the biological role of CES2 and the expression of breast cancer resistance protein (BCRP/ABCG2) in 58 consecutive mCRC patients, who had undergone a first-line CPT-11/5-FU/leucovirin (FOLFIRI) regimen. The expression of these proteins was also examined in a group of synchronous lymph nodes and liver metastases. Furthermore, all samples were revaluated for TS and Topo-I expression. High expression of CES2, ABCG2, TS and Topo-I was observed in 55%, 56%, 38% and 49% of patients, respectively. There was a significant association between high TS and high ABCG2 expression (*p* = 0.049). Univariate analysis showed that only TS expression significantly impacted on time to progression (*p* = 0.005). Moreover, Cox’ multivariate analysis revealed that TS expression was significantly associated with overall survival (*p* = 0.01). No significant correlation was found between investigated markers expression and clinical response. Topo-I expression resulted in being significantly higher in liver metastases with respect to the corresponding primary tumors (*p* < 0.0001), emphasizing the role of Topo-I expression in metastatic cancer biology. In primary tumor tissues, CES2 expression tended to be higher than that observed in liver metastasis tissues (*p* = 0.05). These preliminary data may suggest CES2 over-expression as a potential marker of malignant phenotype. In light of these findings, we suggest that Topo-I expression together with TS expression could be associated with metastatic progression of CRC. Further studies are warranted with the aim of evaluating the potential predictive and prognostic role of CES2 and ABCG2 in larger series of patients.

## 1. Introduction

Irinotecan (CPT-11) belongs to the camptothecin class of topoisomerase I (Topo-I) inhibitors [1]. It is activated by 7-ethyl-10-hydroxy-camptothecin (SN-38), which is approximately 100- to 1000-fold more cytotoxic than the parent drug. In combination with leucovorin (LV) and 5-fluorouracil (5-FU) (FOLFIRI regimen), this drug was able to improve the response rate (RR) and survival of metastatic colorectal cancer (mCRC) patients [2].

Mechanisms of resistance to CPT-11 have been identified as decreased Topo-I levels, reduced drug activation via carboxylesterase 2 (CES2) and increased efflux of CPT-11 and SN38 out of the cell by ATP-binding cassette (ABC) transporters [3]. Expression of drug pathway proteins is independent of tumor type. Few data are available on the relative expression of these proteins in CRC [4,5,6]. In a previous study, we observed that high intratumoral thymidylate synthetase (TS) expression seemed not to preclude the clinical activity of FOLFIRI in mCRC patients. Moreover, the clinical outcome of these patients did not differ in relation to Topo-I or TS expression [7].

Metabolism of CPT-11 is controlled by several enzymes, such as carboxylesterases (CES), a family of serine-dependent esterases involved in the metabolism of endogenous lipids and drugs [8]. These enzymes catalyze the hydrolysis of several clinically important anticancer agents administered as pro-drugs. It has been shown that 5-FU significantly induces CES2 both *in vitro* and *in vivo* [8]. In particular, CES2 is highly expressed in colon tumors, playing a key role in the metabolic activation of CPT-11 [9,10]. Recent results have shown that the expression of CES2 is related to tumor staging [11].

The breast cancer resistance protein (BCRP/ABCG2) is the second member of the ABC-transporter superfamily. In several tumors, high expression of this protein results in resistance to anticancer drugs, including topoisomerase inhibitors [12].

In the present study, we evaluated the biological role of the expression of these proteins in mCRC patients treated with a first-line FOLFIRI regimen and in a group of synchronous lymph nodes and liver metastases.

## 2. Results and Discussion

### 2.1. Results

**Figure 1:**
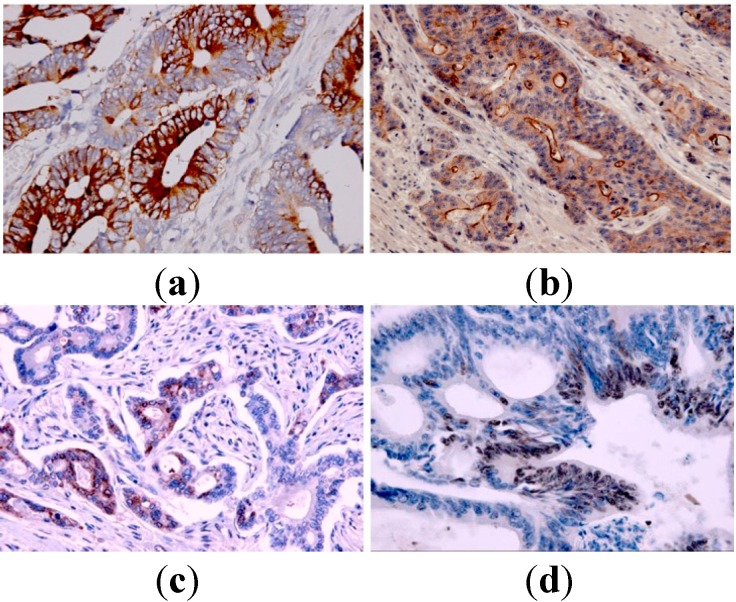
Representative images of immunohistochemical staining for carboxylesterase 2 (CES2), breast cancer resistance protein 2 (ABCG2), thymidylate synthase (TS) and Topo-I in tissues of metastatic colorectal cancer (original magnification 200×). (**a**) Positive cytoplasmic CES2 expression; (**b**) Positive membranous and cytoplasmic ABCG2 expression; (**c**) Positive cytoplasmic TS expression; (**d**) High nuclear Topo-I expression.

We evaluated 58 patients with mCRC who underwent first-line FOLFIRI chemotherapy. The median age of the patients (33 male and 25 female) was 60 years (range: 37–75 years). The location of cancer was the colon in 24 (41%) and rectum in 34 (59%) patients. The median follow-up from commencement of treatment was 24 months (range: 14–28 months). Time to progression (TTP) and overall survival (OS) were nine months (range: 6–10 months) and 18 months (range: 17–21 months), respectively. One patient (1.8%) showed a complete response (CR); 19 (35%) patients showed partial response (PR); 16 (30%) patients had stable disease (SD); and 18 (33%) patients had progressive disease (PD). Four patients were not evaluable for response. All 58 samples were tested with CES, 57 with Topo-I and ABCG2, and 56 samples were tested for TS. Cytoplasmic CES2 overexpression (Subgroups 2 + 3) was present in 55% of tumor tissues. Regarding ABCG2 expression, most positive tumor samples showed a membranous staining and some diffuse cytoplasmic staining. Fifty six percent of tumor tissues showed a strong positivity (Subgroup 2). Over-expression of cytoplasmic TS immunoreactivity was observed in 38% of the tumor tissues. The percentage of nuclear Topo-I positive tumors was 49% (Figure 1).

Expression of these proteins (CES2, ABCG2, TS and Topo-I) was evaluated in relation to the main clinicopathological characteristics of these patients (Table 1). A statistically significant correlation was reported only between CES2 expression and gender (*p =* 0.022 by the Fisher test). Investigation of the relationship among the different protein expressions showed a statistically significant association between TS and ABCG2 expression (*p =* 0.049, by the χ^2^ test) (data not shown). These tumor proteins were investigated by univariate analysis with respect to TTP (Table 2). In univariate analysis, only TS expression significantly impacted on TTP (11 *vs*. six months, respectively; *p =* 0.005 by log rank test). Cox’ multivariate analysis, including CES2, ABCG2 and TS expression, revealed that only TS expression was significantly associated with OS (Table 3), with a hazards ratio of 3.89% (95% confidence interval, CI: 1.26–12.04; *p =* 0.01), but not with the TTP of these patients.

CES2, ABCG2, TS and Topo-I protein expression was evaluated in 19 synchronous liver metastases and 17 synchronous metastatic lymph nodes. Topo-I expression resulted in being significantly higher in liver metastases with respect to the corresponding primary tumors (*p <* 0.0001; by the Kruskal–Wallis test). Indeed, in the primary tumor tissues, CES2 expression tended to be higher than that observed in liver metastasis tissues (*p =* 0.05 by the chi-square test). No correlation was observed between ABCG2 and metastatic sites.

**Table 1:** Patients with high biomarker expression in relation to clinicopathological characteristics.

Characteristics	No. of Pts	High CES2 Expression 2 + 3 No. Cases (%)		High ABCG2 Expression 2 No. Cases (%)		High TS Expression No. Cases (%)		High Topo-I Expression No. Cases (%)	
Gender									
Male	33	23	(70) *	18	(55)	14	(42)	19	(58)
Female	25	9	(36) *	14	(56)	7	(28)	9	(36)
Tumor site									
Colon	24	12	(50)	15	(63)	9	(38)	12	(50)
Rectum	34	20	(59)	17	(50)	12	(35)	16	(47)
Stage									
Primary	38	20	(53)	23	(61)	13	(34)	19	(50)
Recurrent	20	11	(55)	9	(45)	7	(35)	8	(40)
Site									
Liver	20	10	(50)	8	(40)	5	(25)	10	(50)
Other	38	22	(58)	24	(63)	16	(42)	18	(47)
ECOG PS									
0	32	19	(59)	20	(63)	12	(38)	19	(59)
1 + 2	25	13	(52)	12	(48)	9	(36)	8	(32)
Clinical Response									
CR + PR	20	11	(55)	11	(55)	6	(30)	9	(45)
SD	16	9	(56)	10	(63)	7	(44)	6	(38)
PD	18	9	(50)	8	(44)	5	(28)	10	(56)

* *p* = 0.022 by Fisher test. Abbreviations: pts, patients; CR, complete response; PR, partial response; ECOG PS, eastern cooperative group performance status; CES2, carboxylesterase 2; ABCG2, breast cancer resistance protein 2; TS, thymidylate synthetase; Topo-I, topoisomerase-I.

**Table 2:** Univariate analysis considering protein expression with respect to survival in a series of 58 metastatic colorectal cancer patients treated with FOLFIRI.

Characteristics	No. of Pts	Clinical Responses (%) (CR + PR)	Median TTP Months (95% CI)		*p*-Value
CES2					
0 + 1	24	29	11	(8–14)	0.24
2 + 3	29	38	9	(7–11)	
ABCG2					
0 + 1	22	27	10	(7–13)	0.62
2	29	38	9	(3–15)	
TS *					
Low	32	38	11	(9–13)	0.005 **
High	19	32	6	(4–8)	
Topo-I *					
Low	28	32	10	(8–12)	0.58
High	24	38	9	(6–12)	

* Cut-off: median value of the series; ** *p* = 0.005 by log rank test. Abbreviations: pts, patients; CR, complete response; PR, partial response; TTP, time to progression; CES2, carboxylesterase 2; ABCG2, breast cancer resistance protein 2; TS, thymidylate synthetase; Topo-I, topoisomerase-I.

**Table 3:** Cox multivariate analysis of OS in a series of 58 metastatic colorectal cancer patients.

Characteristics	OS Hazards Ratio (95% CI)	*p-*Value
CES2 (2 + 3 *vs.* 0 + 1)	0.74 (0.26–2.13)	0.58
ABCG2 (2 *vs.* 0 + 1)	0.54 (0.19–1.54)	0.25
TS (high *vs*. low) *	3.89 (1.26–12.04)	0.01

* Median value. Abbreviations: CI, confidence interval; OS, overall survival; CES2, carboxylesterase 2; ABCG2, breast cancer resistance protein 2; TS, thymidylate synthetase.

**Figure 2:**
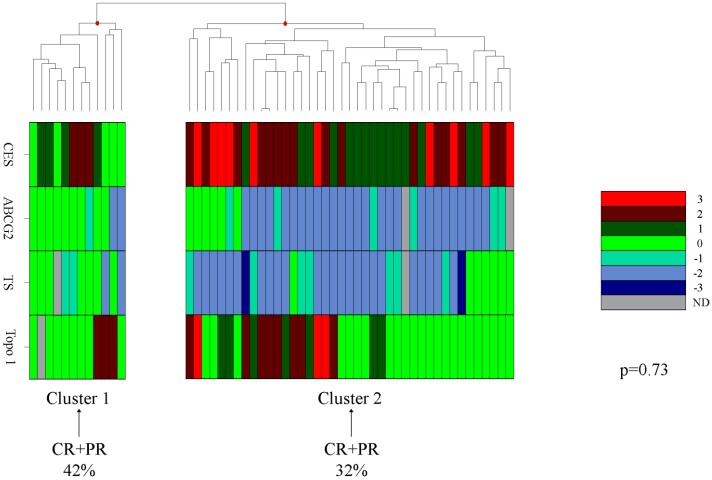
Hierarchical clustering from CES2, ABCG2, TS and Topo-I protein expression performed in 53 metastatic colorectal cancer (mCRC) patients.

Unsupervised hierarchical clustering from CES2, ABCG2, TS and Topo-I protein expression was then applied in 53/54 patients evaluable for clinical response. The patients were separated into Cluster 1 (CR + PR = 42%) and Cluster 2 (CR + PR = 32%) based on the expression of these proteins in relation to clinical outcome. The two groups showed a comparable percentage of clinical response (*p* = 0.73) (Figure 2).

### 2.2. Discussion

CPT-11 in combination with 5-FU/LV is widely used in mCRC patients. In this study, we examined the subcellular expression of CES2 and ABCG2 by immunohistochemistry in a well-selected consecutive series of mCRC patients treated with the FOLFIRI regimen and investigated their relation to clinical outcome. The expression of TS and Topo-I was revaluated on the same tumor samples. The association between TS and Topo-I expression and clinicopathological characteristics has been previously reported [7]. Our data demonstrated that clinical response and survival of mCRC patients treated with the FOLFIRI regimen correlated with TS expression. These data confirm the results by Maithel *et al.* [13] reporting a correlation between TS expression and a lower relapse-free survival in mCRC patients. In the present study, 55% of advanced tumors demonstrated high cytoplasmic expression of CES2. CES2 overexpression has been associated with increased irinotecan activity *in vitro*, even if its clinical relevance is less well determined [14]. Recently, Uchida *et al*. [1] showed that CES2 RNA expression in neuroblastoma was correlated with protein levels of CES2 and was higher in patients with advanced disease. We found a significant correlation between CES2 expression and gender, but no relationship with clinicopathological characteristics.

ABCG2, the second member of the G subfamily of the ATP-binding cassette efflux transporter superfamily, has been the subject of several studies. ABCG2 plays an important role in multidrug resistance, but its expression is controversial in different tumor types, including colorectal cancer [15]. ABCG2 expression in CRC and its relationship with invasion and metastasis is still not clear. We found high expression of ABCG2 in 56% of patients in the absence of an association with clinicopathological characteristics. Moreover, ABCG2 tumor expression did not affect the short- and long-term clinical outcome of these patients. These findings were partly confirmed by the study of Wang *et al*. [16], who showed a lack of correlation between cytoplasmatic ABCG2 staining and clinical outcome [17]. TS is a key enzyme for DNA synthesis and the cellular target of 5-FU for the medical treatment of CRC. Several studies have reported that increased TS levels in tumors are associated with resistance to 5-FU, and its expression has been shown to be an independent prognostic factor in several cancers [17]. Moreover, for CRC, TS has been shown to be a prognostic marker for survival rather than a predictive marker for chemotherapy response [18]. No significant differences in TS expression were found in relation to clinicopathological characteristics, in agreement with other authors [19,20].

In our cohort of mCRC patients, multivariate analysis confirmed literature data, which reports TS as an independent variable for OS when the expression of this protein was categorized as one variable [21]. Cluster analysis identified two subgroups of patients characterized by a low expression of the proteins in Cluster 1 compared to Cluster 2. In Cluster 2, we observed a low expression of ABCG2 and TS and a high expression of Topo-I and CES2 expression. The patients included in Cluster 1 showed a response to treatment of 42% compared to 32% of the patients in Cluster 2. Although the results are not significant, they suggest that low expression of these proteins could be associated with a favorable clinical response.

In our series of 19 patients with primary tumors and corresponding synchronous liver metastases, as we had previously demonstrated, a higher expression of Topo-I was observed in synchronous liver metastases than in the corresponding primary tissues, showing an association with the acquisition of a metastatic phenotype. Intriguingly, a statistical trend was also observed for CES2 expression. CES2 immunoreactivity was higher in the primary tumor tissues than in the corresponding metastatic tissues. This observation may suggest that CES2 over-expression in primary tumor tissues could be a potential marker of the malignant phenotype. Evaluation of this protein in stage I, II and III lesions and in metachronous metastases may be essential to confirm this hypothesis.

## 3. Experimental Section

### 3.1. Patients

Fifty eight consecutive specimens of mCRC were obtained retrospectively from the Department of Pathology at the National Cancer Research Centre (NCRC), Istituto Tumori “Giovanni Paolo II” of Bari, Italy, between 1997 and 2003. Patients received a first-line FOLFIRI chemotherapy regimen, and most of them were treated within a phase III clinical trial [22]. The retrospective use of human colon samples was approved by the Institutional Review Board of our Institute (NCRC; project number: 28; 2004). Before undergoing routine surgery, all patients signed an informed consent form authorizing the NCRC to utilize their removed biological tissue for research purposes according to ethical standards. Response to treatment was re-evaluated according to Response Evaluation Criteria in Solid Tumors (RECIST) 1.1 criteria [23].

### 3.2. Immunohistochemistry (IHC)

Expression of CES2 and ABCG2 was detected in 58 samples of mCRC by IHC. The expression of TS and Topo-I was also revaluated on these samples [7]. The corresponding hematoxylin and eosin stains were assessed to confirm the diagnoses of these patients. Moreover, 19 patients with primary tumors and corresponding synchronous liver metastases, of whom 17 also had synchronous metastatic lymph nodes, were examined for CES2, ABCG2, TS and Topo-I protein expression. Sections of 4-μm thickness were cut from formalin-fixed and paraffin-embedded histological blocks, and these were immunohistochemically stained using standard immunoperoxidase techniques, as previously described [24]. The sections were deparaffinized in xylene, rehydrated through a graded ethanol series and pretreated with 0.01 M sodium citrate buffer at pH 6.0 in a water bath. After endogenous peroxidase activity blocking with 0.3% H_2_O_2_ buffer solution, sections were incubated at 4 °C overnight with mouse monoclonal antibody for CES2 (G5, 1:100 dilution, Santa Cruz Biotech., Dallas, TX, USA), mouse monoclonal antibody for ABCG2 (clone BXP-21, 1: 50 dilution, Abcam Company, Cambridge, MA, USA), mouse monoclonal antibody for TS (TS106, 1:100 dilution, Santa Cruz Biotech.) and NCL-TOPO I for Topo-I (clone 1D6, 1:50 dilution, Novocastra, Leica Biosystems, Milano, Italy).

For all of the biomarkers, the bound antibody was visualized with 3,3-diaminobenzidine tetrahydrochloride (DakoCytomation, Glostrup, Denmark), and the sections were then counterstained with Mayer’s hematoxylin. Known positive controls were included in each staining run. Omission of the primary antibody was used as a negative control. When a section was either uninformative or lost, a case was judged as “not assessable” in the statistical analysis. Immunohistochemical analysis of CES2, ABCG2, TS and Topo-I was based on subcellular localization of the proteins. Protein expression analysis for nuclear and cytoplasmic antigens was quantified by counting positive cells in 3 to 5 representative areas for each section at 20× magnification and expressed as a percentage of positive cells/section. The intensity of staining was scored as 0 (negative), 1 (weak), 2 (medium) or 3 (strong).

The cytoplasmic expression pattern of CES2 in tumors was scored as 0 (no positive cells), 1 (1%–25% of positive cells), 2 (26%–50% of positive cells) or 3 (51%–100% of positive cells). For ABCG2 expression, membranous and cytoplasmic staining was observed. The samples were scored as 0 (no positive cells), 1 (≤10% of positive cells) or 2 (>10% of positive cells) [25,26]. In the statistical analysis, CES2 and ABCG2 expression were classified into two subgroups: 0 + 1 (negative cells) and 2 + 3 (positive cells) for CES2; and 0 + 1 (negative cells) and 2 (positive cells) for ABCG2. The median value of positive cells was used as the cut-off to group cases into two categories of negative (≤median) and positive (>median) cytoplasmic and/or nuclear expression. For TS expression, the cases were classified as positive when cytoplasmic immunoreactivity was present in >30% of tumor cells (median value: 30). TS positive staining was revealed as a dark brown and granular cytoplasm signal. For Topo-I expression, the cases were classified as positive when nuclear immunoreactivity was present in >1.5% of tumor cells (median value: 1.5). Topo-I expression was revealed as a specific dark brown nuclear signal. The slides were examined independently by two observers blinded to both clinical and pathological data.

### 3.3. Statistical Analysis

Statistical analyses were performed using SPSS 14.0 statistical software (SPSS Inc., IL, Chicago, CA, USA). Objective response (OR; CR + PR) was reported as its relative rate with a 95% confidence interval (95% CI). Levels of statistical significance were evaluated with data by using the chi-square test or Fisher’s exact test to determine FOLFIRI clinical responsiveness and protein expression levels. The Kruskal–Wallis test was performed to compare protein expressions in the analysis. The proteins that showed a trend toward statistical significance were analyzed by logistic regression to adjust for clinical characteristics known to be associated with 5-FU/CPT-11 sensitivity. The Student’s *t*-test was used to compare means of variables for all of the groups. Median TTP and OS and 95% CI were estimated using the Kaplan–Meier method and compared by the log rank test. TTP and OS were determined from the date of the first cycle of chemotherapy until progression, death or last follow-up.

Cox regression analysis was performed to assess the predictive power on clinical outcome of the proteins CES2, ABCG2, TS and Topo-I assessed by IHC. Two-sided tests were computed, and all statistical differences were considered significant at the level of *p <* 0.05.

Cluster analyses were generated by the unsupervised system.

## 4. Conclusions

In light of these findings, we suggest that Topo-I expression together with TS expression [14] could be associated with metastatic progression of CRC. TS expression correlates with clinical outcome of mCRC patients treated with a first-line FOLFIRI regimen. However, the low number of patients limits the statistical importance of the study overall, even if, in our opinion, the confirmation of the inverse relationship between TS expression and clinical outcome is relevant. Further studies are warranted with the aim of evaluating the potential predictive and prognostic role of CES2 and ABCG2 in larger series of patients.
